# Influence of Structure on Electronic Charge Transport in 3D Ge Nanowire Networks in an Alumina Matrix

**DOI:** 10.1038/s41598-019-41942-3

**Published:** 2019-04-01

**Authors:** Nirat Ray, Nikita Gupta, Meghadeepa Adhikary, Nikolina Nekić, Lovro Basioli, Goran Dražić, Sigrid Bernstorff, Maja Mičetić

**Affiliations:** 1 School of Physical Sciences, Jawaharlal Nehru University, Delhi, 110067 India; 2 Department of Materials Science and Engineering, Indian Institute of Technology, Delhi, 110016 India; 3 Ruđer Bošković Institute, Bijenička cesta 54, 10000 Zagreb, Croatia; 4 National Institute of Chemistry, 1000 Ljubljana, Slovenia; 5 Elettra-Sincrotrone Trieste, 34149 Basovizza, Italy

## Abstract

We demonstrate formation of material consisting of three-dimensional Germanium nanowire network embedded in an insulating alumina matrix. A wide range of such nanowire networks is produced using a simple magnetron sputtering deposition process. We are able to vary the network parameters including its geometry as well as the length and width of the nanowires. The charge transport in these materials is shown to be related to the nanowire surface per unit volume of the material, *α*. For low values of *α*, transport is characterized by space charge limited conduction and a drift of carriers in the extended states with intermittent trapping-detrapping in the localized states. For large values of *α*, charge transport occurs through hopping between localized electronic states, similar to observations in disorder-dominated arrays of quantum dots. A crossover between these two mechanisms is observed for the intermediate values of *α*. Our results are understood in terms of an almost linear scaling of the characteristic trap energy with changes in the nanowire network parameters.

## Introduction

Nanoscale building blocks, such as nanocrystals, nanotubes, wires, and rods, and the corresponding three dimensional nanostructures have been studied intensively primarily due to the unique properties arising from an interplay of low dimensionality and quantum effects as well as the potential for direct integration into nanoscale devices^1^. Such network structures can be thought of as artificial solids wherein the properties of the solid are dictated not by the atomic composition of the material but by the properties of the nanoscale building blocks^2^. Semiconductor nanowire based structures have shown promise for applications in a host of fields ranging from optoelectronics^3–6^ to bioelectronics and chemical sensors^7–10^ and high-performance thermoelectrics^11^. Metallic and semiconducting nanowire networks have been typically achieved by spatial arrangements of particles into lines and arrays through colloidal dispersions in liquid crystals, field-induced dipole interactions, or the physical/chemical confinement of a colloidal suspension within templates^12–22^. However, such confinement approaches often require high-resolution lithography for defining patterns at a nanometer scale, and thereby limit the flexibility in network design, and/or often involve insufficient connectivity between the adjacent particles. Furthermore, applications to any electronic devices would require an understanding of the conduction mechanism in the networks and identification of parameters to tune the mechanisms.

In this paper, we demonstrate a material consisting of continous three-dimensional (3D) germanium (Ge) nanowire networks embedded in an insulating alumina matrix produced by magnetron sputtering, and we explore its structural and electronic properties. The networks have body centred tetragonal arrangement of their nodes and highly tuneable unit cell parameters. By varying primarily the Ge concentration, we easily synthesize networks having a wide range of structural parameters including the length and width of nanowires, the geometry of the network nodes and consequently different nanowire surface area per unit volume which is found important for charge transport properties. Beside the highly tunable structural properties of the networks, transport studies suggest a strong correlation between the charge transport mechanism and the structural parameters of the network. The important parameter is found to be the nanowire surface per unit volume of the material, *α*. For low values of *α*, charge transport is characterized by the drift of carriers in the extended states with intermittent trapping-detrapping in the localized states. For large values of *α*, transport occurs through hopping between localized electronic states, similar to observations in arrays of quantum dots. Both mechanisms are seen to coexist for intermediate values of *α*. Our results are understood in terms of an almost linear scaling of the characteristic trap energy (*E*_*t*_) with changes in the nanowire network parameters.

The nanowires obtained by vapor liquid solid (VLS) method are extensively studied, and they show many exciting properties like light trapping abilities, enhanced photoresponse and generally large improvement of solar-cell devices^23–27^. Based on these studies we believe that our system could show some similar features. Due to the large difference in refraction index between Ge and alumina combined with tunable geometry of the nanowire network we believe that the material has enhanced light trapping abilities. Another interesting feature that we could expect is decreased response time in application in Ge-based photodetectors. Finally, Ge shows very strong confinement, resulting in large increase in Ge bandgap with reduction of Ge size (radius). This fact could be used to control the transparency of the nanowire network films.

## Experimental

The networks of Ge nanowires are formed by a self-assembly process in amorphous aluminum dioxide (Al_2_O_3_) matrix by magnetron sputtering - KJLC CMS-18 deposition system. Pure Ge (99.995%) and Al_2_O_3_ (99.995%) were co-deposited on Si(111) substrate at 500 °C and 300 °C. The sputtering powers of Ge and alumina targets were tuned to change the atomic percentage of Ge in the films. The deposition temperature was used to tune the disorder in the films. The deposition parameters are given in Table 1. This technique has been previously used to form Ge and Ge/Si quantum dots^28–32^, embedded in different dielectric matrices. The matrices serve to electrically isolate the nanowires and also assist in formation of self assembled structures. We use Transmission electron micrography (TEM), grazing incidence small-angle X-ray scattering (GISAXS) and X-ray diffraction (XRD) to study the geometrical properties, connectivity of the network and their crystalline structure. GISAXS was measured at synchrotron Elettra, Trieste using the photon energy of 8 keV. TEM was performed using JEOL 2010 F microscope, operated at 200 kV and equipped with a field-emission gun and a high-angle annular dark-field detector (HAADF) for Z-contrast imaging; XRD is performed using a D5000 thin-film diffractometer. The electrical characterizations of two terminal sandwich structures are carried out using Keithley 2611 sourcemeter and Keithley 6485 picoammeter. The temperature dependence measurements are done inside a CTI-Cryogenics close cycle cryostat and temperature is controlled with a Lakeshore temperature controller.

**Table 1:** Deposition and structural parameters of different films.

#	*T*_*d*_ °C	P_*Ge*_ *W*	${{\boldsymbol{P}}}_{{\boldsymbol{A}}{{\boldsymbol{l}}}_{{\bf{2}}}{{\boldsymbol{O}}}_{{\bf{3}}}}$ *W*	*a* *nm*	*c* *nm*	*r* *nm*	*l* *nm*	*α* *nm*^−1^
S1	500	6.5	300	9.5	7.3	1.2	9.9	0.4
S2	500	40	300	5.6	3.3	1.1	5.2	1.3
S3	500	60	300	5.0	2.8	1.0	4.5	1.6
S4	500	40	150	4.8	1.9	0.9	3.9	1.9
S5	300	60	300	3.2	2.5	0.7	3.4	2.3

*T*_*d*_ is the deposition temperature, P_*Ge*,*Al*2*O*3_ are the deposition powers for Ge and Al_2_O_3_, *a* and *c* are the moduli of the basis vectors ***a***_1,2_ and ***a***_3_ respectively, *a* = |***a***_1_|, ${\rm{c}}={\boldsymbol{|}}{{\boldsymbol{a}}}_{3}^{z}{\boldsymbol{|}}$, and *r* is the nanowire radius, and *l* is their length. *α* measures the the ratio of the nanowire surface to unit cell volume (*a*^2^*c*).

## Structural Properties

**Figure 1:**
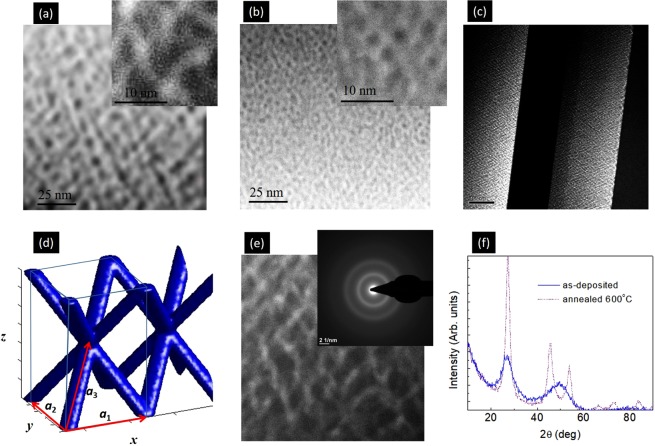
(**a**,**b**) TEM cross-sectional images of networks S1 and S2. (**c**) Cross section of the entire film S1. (**d**) Model of the geometry of the formed nanowires lattice; ideal structure is assumed. The arrows indicate basis vectors of the lattice. (**e**) TEM cross-sectional image of S1 with better resolution. The inset shows selected area electron diffraction (SAED) image. (**f**) XRD spectra of film S2 after deposition (full blue line) and after annealing (dashed violet line). The peaks positions correspond to crystalline Ge.

Figure 1 demonstrates the main structural properties of the films. From the cross-sectional TEM images demonstrated in Fig. 1(a and b), we can see the 3D nanowire network and the connectivity that is formed during the deposition process. As visible from the TEM images, the geometrical properties of the networks depend strongly on the deposition parameters, so the networks with very different parameters can be produced. The 3D nanowire network occurs during the whole film thickness, with the nearly constant ordering properties. This is visible in Fig. 1(c) which shows the cross section of the entire film.

The structure of the films is the best described by unit cell of body centered tetragonal (BCT) lattice, in which the lattice sites are connected by Ge nanowires, as shown in Fig. 1(d). So, in the ideal case, the nanowires are expected to form a 3D mesh in which the Ge nanowires are directed towards the nodes of BCT lattice defined by basis vectors ***a***_1_ − ***a***_3_, as shown in Fig. 1(d), where, $|{{\bf{a}}}_{1}|=|{{\bf{a}}}_{2}|=a$ and $|{{\bf{a}}}_{3}^{z}|=c$. This is of course the ideal case; the real lattice deviates more or less from the ideal one depending on the deposition conditions. One real case is shown in Fig. 1(e). These disorder properties will be the topic of our next paper. Here we focus on the main geometrical properties of the network, i.e. the parameters of the ideal unit cell. The main features of the growth mechanism that induces formation of the 3D networks is described in ref.^28^. It is combination of diffusion mediated nucleation and the influence of surface morphology on the nanowire growth. It is important to note that the nanowiress form in fully amorphous systems, so the strain effects are not efficient here. Similar mechanism governs formation 3D ordered arrays of Ge quantum dots in amorphous alumina or silica matrices^29–31,33^. As we mentioned above, the structure of the as-deposited films is fully amorphous, i.e. both Ge nanowires and alumina matrix are amorphous. That follows from the selected area electron diffraction (SAED) measurements of the films cross section (inset of Fig. 1(e)), high resolutionTEM images of the film and also from the standard XRD measurements performed at grazing incidence angle (Fig. 1(f)). Such amorphous structure always forms in films consisting of Ge quantum dots or nanowires in alumina or silica matrices deposited by magnetron sputtering deposition^29,33^. Crystallization of the films may be induced by annealing of the films at 600 °C, as visible from the dashed XRD curve shown in Fig. 1(f). The formed Ge crystals are small in size and have randomly oriented grains. The nanowire structure stays stable for annealing temperatures up to 700 °C. For higher temperatures destruction of the 3D network lattice occurs.

**Figure 2:**
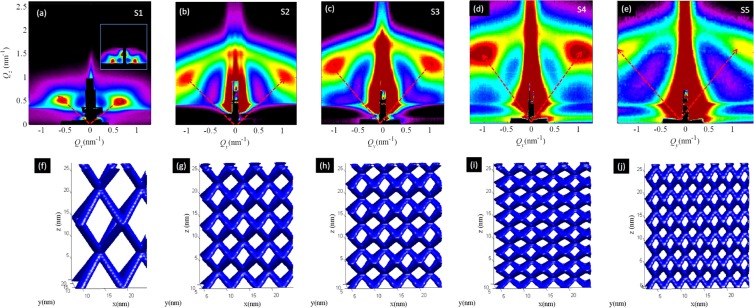
(**a**–**e**) GISAXS maps of the films S1-S5, having different nanowire length and arrangement (see Table 1) and (**f**–**j**) corresponding structure simulations (ideal structure and no disorder, is assumed) obtained by GISAXS analysis. The inset in panel (a) is simulation of the experimentally measured GISAXS map obtained by using of the fitting parameters. The arrows indicate positions of the Bragg peaks.

GISAXS has proven to be a powerful tool in analyzing the spatial organization and size distribution of buried nanoscale materials^34–36^. The ordering properties of the nanowire networks are clearly visible as strong reflections or Bragg spots in the GISAXS maps of the films S1-S5 (indicated by arrows in Fig. 2(a–e)). Their positions in the reciprocal (*Q*) space are related to the geometry of the formed nanowire mesh. Very different positions demonstrate large difference in the mesh parameters, as will be demonstrated in the following text.

Detailed structural parameters including arrangement, size properties and their statistical distributions are found by numerical analysis of the GISAXS data^36^. The basic parameters obtained by the numerical analysis of the films investigated here are given in Table 1. An example of the simulated GISAXS map, using the parameters obtained by fit is shown in panel of Fig. 2(a). From the parameters it is clear that the nanowire length and basis vector parameters can be tuned over a large range by varying the deposition conditions. This effect is well visible in Fig. 2(f–i) where the ideal structures of the films are simulated using the data from the GISAXS analysis. For samples S1, S2 and S3, deposited at the same deposition temperature of 500 °C, we find that the length of the nanowires (*l*) and the nanowire radius (*r*) decreases with increasing the sputtering power of Ge target. This results in a higher density of mesh nodes for a higher Ge power. Networks with the smallest nanometer radius of 0.7 nm were formed by using a lower deposition temperature and high Ge power. Intermediate densities and nanowire radii were obtained by weaking the alumina power as well. We can also tune the nanowire length *l* in a large range; For the samples presented here it ranges from 3.4 nm to 9.9 nm.

To compare the properties, we have calculated the nanowire surface per unit volume of the material for each film. This value is expressed by the parameter, *α* given in Table 1. This value is clearly lowest for sample S1 having the smallest Ge concentration and largest nanowire length. We have studied samples with *α* ranging from 0.4 nm^−1^, to 2.3 nm^−1^, as listed in Table 1.

Similar nanowire networks is possible to grow also with some other oxide matrices (unpublished data). However, the best ordering of the nanowires is achieved with alumina as a matrix. It also reduces oxidation of Ge as found by other authors as well^37^. It is reasonable to expect that some Ge on the surface of nanowires oxidase, however the rest of Ge is non-oxidized as visible from the XRD measurements.

## Charge Transport Properties

**Figure 3:**
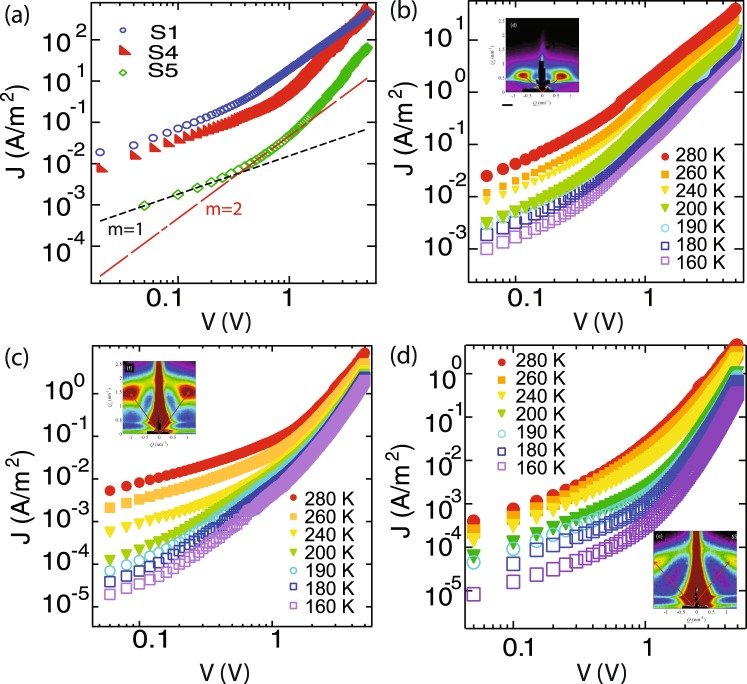
(**a**) Log-log plot of the current density-voltage (J-V) characteristics for devices S1 (blue open circles), S4 (red filled triangles) and S5 (green open diamonds), at room temperature. Black Dashed lines show fits to ohmic conduction and red dashed lines represent fits to Mott-Gurney law describing space charge limited conduction (SCLC) as described by Eq. 1. (**b**–**d**) J-V characteristics at different temperatures for samples S1, S4 and S5 respectively.

Figure 3(a) shows the current density-voltage (J-V) characteristic for three nanowire networks: S1 (deposited at high temperature with low Ge target power and high alumina target power, $\alpha =0.4$), S4 (deposited at same temperature but higher Ge power and lower alumina power, $\alpha =1.9$) and S5 (deposited at lower temperature with high Ge and alumina power, $\alpha =2.3$), at room temperature. Results for samples S2 and S3 are similar to S4. For all three samples, at low bias voltages, we observe linear characteristics from the application of a positive bias on the gold electrode, suggesting the formation of an ohmic contact. The resulting current density, J, is then directly proportional to the concentration of thermally generated carriers, *n*_0_, $J=\mu q{n}_{0}V/d$. Here *μ* is the mobility, *q* is the electron charge, *V* is the applied voltage and *d* is the length of the channel, and for the sandwhich structures studied here, corresponds to the thickness of the measured films. As the bias is increased, the J-V characteristics become increasingly nonlinear, and over a finite region, the current density varies quadratically with the applied bias, consistent with space charge limited conduction (SCLC)^38^. In this region, the injected current density is given by the Mott-Gurney law^38–42^:

1 $$J=\frac{9}{8}\mu \epsilon {\epsilon }_{0}\frac{{V}^{2}}{{L}^{3}}$$

The charge carrier mobility can therefore be directly extracted from the quadratic part and the background density from the linear regime. Extracted room temperature carrier mobilities range from 10^−5^–10^−6^ cm^2^/Vs for all samples.

We find deviations from the Mott-Gurney law at higher bias voltages. The deviations are seen to be strongly temperature dependent for all three samples (Fig. 3(b–d)). This is not surprising as traps or localized electronic states are expected to play an important role in determining the current density-voltage characteristics particularly in disordered materials, and the Mott-Gurney law typically describes transport in a trap free material with a field independent mobility^39,40^. The influence traps have been extensively studied by assuming different energetic distributions of localized states: exponential, Gaussian, uniform and discrete levels, has been examined extensively in literature, particularly for the case of organic semiconductors^40,41^. If we assume a distribution of such states, with constant mobility, the resulting current density (J)-voltage(V) characteristics are given by:

2 $$J={q}^{1-l}\mu N{\frac{2l+1}{l+1}}^{l+1}{(\frac{l}{l+1}\frac{{\epsilon }_{s}{\epsilon }_{0}}{{H}_{t}})}^{l}\frac{{V}^{l+1}}{{d}^{2l+1}}$$

where, *H*_*t*_ is the density of traps, ${\epsilon }_{0}$ is the permittivity of free space, ${\epsilon }_{s}$ is the dielectric constant of the material, *μ* is the carrier mobility, *N* is the density of states in the relevant band and *d* is the sample thickness. The power law exponent for the voltage dependence, $l={T}_{c}/T$, and is related to the characteristic trap temperature *T*_*c*_ in the exponential distribution of trap states, and *T* is the measurement temperature. We can further associate the energy E_*t*_ = *kT*_*c*_ with the traps.

**Figure 4:**
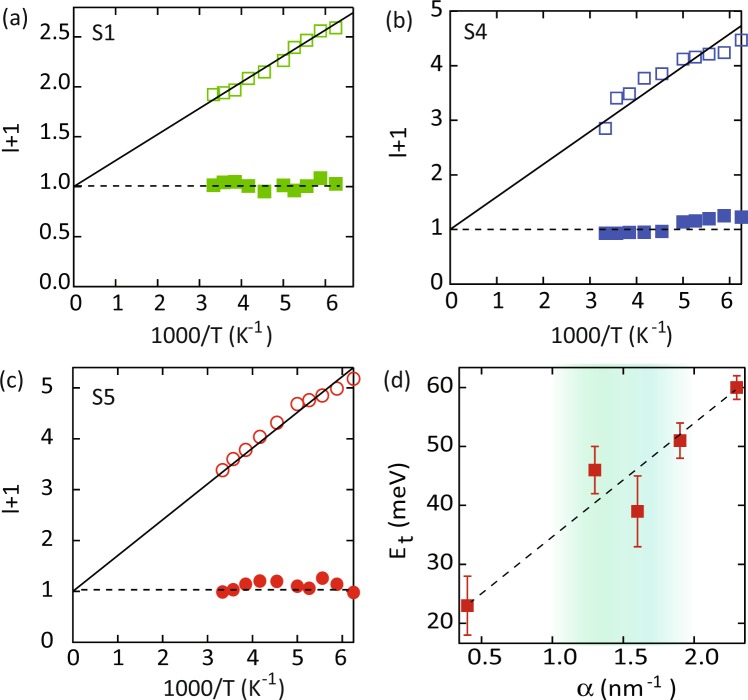
Power law exponent, $l+1$ for the current density-voltage (J-V) characteristics in the low bias (filled markers) and high bias (open markers) regime, for samples (**a**) S1, (**b**) S4 and (**c**) S5 respectively. At low bias, Ohmic behavior is observed corresponding to $J\propto V$. In the high bias regime, solid lines are fits to $l={T}_{c}/T$, where *T*_*c*_ is the characteristic trap temperature for an exponential distribution of traps. (**d**) Characteristic trap energies (*kT*_*c*_) as a function of the nanowire surface to volume parameter *α* defined previously. The dashed line serves as a guide to the eye.

We find that this model works very well for our nanowire networks. Figure 4(a–c) show the exponent *l* + 1 as a function of inverse temperature for three samples S1, S4 and S5 respectively. At low bias voltages (filled markers), $l+1=1$, consistent with Ohmic behavior. At higher bias voltages (open markers), we extract the characteristic energies of the trap distribution (*kT*_*c*_) from the slope of a log-log plot of the J-V characteristics by fitting to Eq. 2. Figure 4(d) shows the characteristic trap energies (*kT*_*c*_) as a function of the surface to volume parameter *α* defined previously. The dashed line serves as a guide to the eye. As mentioned earlier, samples S2 (*α* = 1.3) and S3 (*α* = 1.6) show behaviour similar to S4. Characteristic trap distribution energies are extracted similarly for these samples as well. Interestingly, we find that the trap energies scale almost linearly with *α*. Shallow traps or low energy values are associated with longer nanowires or correspondingly smaller nanowire surface to unit cell volume ratios. Deeper trap energies are observed for shorter nanowires and correspondingly larger nanowire surface.

The ohmic region for S4 is seen to be strongly temperature dependent, whereas it is largely temperature independent for S3. In the classical analysis of charge transport in solids by Lampert^43^, an Ohmic current is typically attributed to the presence of a background carrier density due to (unintentional) doping. From the voltage at which a crossover from ohmic to SCLC is observed, we may extract this carrier concentration, *p*_0_. From the temperature dependence of *p*_0_, a 60 meV activation energy for the doping process can be extracted. It is interesting to note that this value matches with the binding energy of Al. It is then conceivable that the Al from the matrix may act as an acceptor impurity for the Ge nanowires. It was recently also pointed out that Ohmic currents can also result from diffusion of charge carriers from the contacts into the semiconductor^44^. However, if this was the dominant mechanism, similar temperature dependent results would have been observed for all nanowire networks, which is not the case.

**Figure 5:**
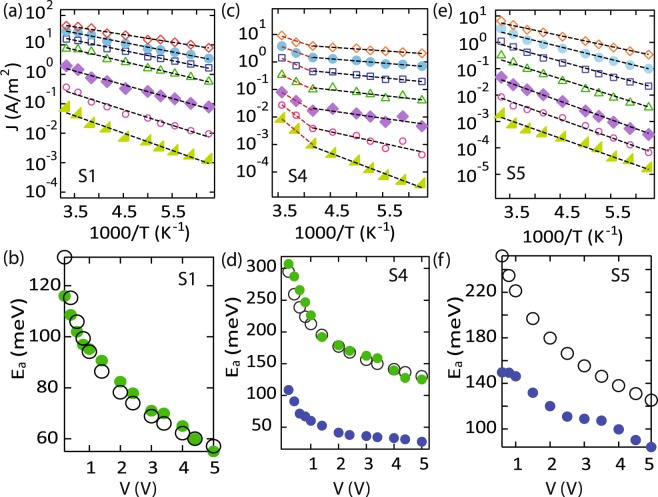
Current density as a function of inverse temperature for different values of the applied voltage, V for samples S1 (**a**), S4 (**c**) and S5 (**e**). The current is fit to a simple Arrhenius form $J\sim \exp (\,-\,{E}_{a}/kT)$, where *E*_*a*_ is the activation energy, *k* is the Boltzmann’s constant and *T* is the temperature. (**b**,**d** and **f**) Show the activation energies, *E*_*a*_ (in meV) extracted from fits to the data in (**a**,**c** and **e**) respectively, as a function of applied voltage, V.

It is important to note that current conduction in solids with localized states can be complicated in general, and other mechanisms such as Schottky emission, Fowler-Nordheim injection, Poole-Frenkel conduction etc. may also play a role. While a majority of these effects can be ruled out from the shape of the J-V characteristics, in order to rule out tunneling effects, we focus on the temperature dependence of the current at fixed bias voltages. On studying the temperature dependence of each of the samples, we observe important differences as discussed below. Figure 5(a) shows the current density (J) as a function of inverse temperature (1000/T) for sample S1. We find that the current density is simply activated and can be described by a simple Arrhenius form $J\sim \exp (\,-\,{E}_{a}/kT)$, where *E*_*a*_ is the activation energy and *k* is the Boltzmann’s constant. The activation energy decreases with an applied voltage as shown in Fig. 5(b). Within the trap model described above, the current would be almost temperature independent at a crossover voltage *V*_*c*_. Thus, it is enough to take the J-V data for two or three temperatures in appropriate range, prepare log-log plots, extrapolate the power law to determine the crossover voltage *V*_*c*_. In this picture, the temperature dependence of the current is expected to be simply activated and given by:

3 $${E}_{a}={E}_{t}\,ln(\frac{{V}_{c}}{V})$$

Red filled markers in Fig. 5(b) show the activation energies extracted from fits to the experimental data, and black empty markers depict the activation energies predicted by the trap model, as described by Eq. 3. The excellent agreement between these results, suggests that the transport for this sample is governed by drift of charge carriers under the influence of an exponential distribution of traps or localized states.

For samples S2-S4, we find that the data cannot be fit to a single activation energy. However, we may extract two activation energies by fitting the the data to a sum of two simply activated processes, as shown in Fig. 5(c) for sample S4. The crossover between the two activation energies occurs at a temperature of ~200 K for S4. The crossover temperature is much lower for sample S2 (130 K) and comparable for sample S3. Interestingly, the high temperature activation energy matches well with the predictions of the trap model, as shown in Fig. 5(d). Therefore, we conclude that the current in the high temperature region (>200 *K*) is due to the drift of carriers in the extended states with intermittent trapping-detrapping in the localized states. As we go to low temperatures, the population in the extended states becomes negligible, and conduction may then take place through the tunneling or hopping of carriers between localized electronic states, resulting in a weaker temperature dependence. Eventually signatures of variable range hopping may also be observed by going to lower temperatures. This effect is absent from sample S1 presumably due to the lower characteristic trap energy, and consequently availability of shallow trap states. The effect may become prominent in S1 on going to lower temperatures.

Finally, the temperature dependence of sample S5 can also be fit to a simply activated process as shown in Fig. 5(e). The activation energy decreases almost linearly with applied voltage as shown in Fig. 5(f). However, the experimental results (filled markers) do not agree with those predicted by the trap model discussed above. This observation then suggests that for this configuration, the charge carriers are strongly localized, and conduction occurs only through hopping between nearest neighbor sites. This is further supported by the deeper trap energies extracted for this configuration. Hopping conduction between localized states is often reported for arrays of quantum dots^45–47^. It is then conceivable that large values of the parameter *α* indicate the collapse of the system into a set of discrete energy levels similar to those reported in disorder dominated quantum dot assemblies.

## Conclusion

In conclusion, we have investigated the structural and electronic properties of three-dimensional Ge nanowire networks embedded in an insulating alumina matrix, synthesized by magnetron sputtering. A large range of 3D Ge nanowire meshes are demonstrated. They differ by the length and width of nanowires and by the geometrical properties of the mesh. We find that the conduction mechanism can be tuned by varying the deposition parameters, and the resulting structural changes can be easily characterized by small angle X-ray scattering measurements. The changes in the network parameters are best characterized by the nanowire surface per unit volume, *α* which has been varied from 0.4 to 2.3. From transport studies, we find thermally generated carriers showing ohmic behavior at low bias voltages. At higher bias voltages, as carrier injection becomes dominant, the transport mechanism is characterized by SCLC under the influence of a distribution of traps or localized states. For low values of the parameter *α*, this model describes exactly the conduction mechanism. As *α* increases, the conduction mechanism crosses over to a combination of drift of carriers in the extended states with intermittent trapping-detrapping in the localized states and hopping between localized states to only hopping between localized states. For large values of *α* the system starts to mimic the behavior expected for quantum dot arrays rather than nanowire networks. Our results then suggest that the characteristic trap energy scales almost linearly with *α*, and changes in the conduction mechanism are completely driven by this energy scale. Our results thus demonstrate the use of these materials as tunable testbeds for exploring the physics of disordered materials, and could potentially drive further research into nanowire based artificial solids with the potential for rich physics and novel device applications.
